# Where Is the Fault?

**Published:** 1879-04

**Authors:** R. L. Cochran


### WHERE IS THE FAULT?

BY DR. R. L. COCHRAN.

*Read before the Iowa State Dental Society, July 1878.*

*Mr. President and Fellow-Practitioners:*

I have selected a subject for your consideration to-day, which I have chosen to name "Where is the Fault?" and while I may take strong grounds against the individual ideas of some of my professional brethren, I hope they will not regard it as personal.

The Dental profession, gentlemen, at the present time seems to be in a whirlpool of excitement over the late declaration made by men high in professional attainments and who have been successful practitioners and is it any wonder that the profession should be in such condition when men of no ordinary intelligence as Flagg, Chase and others, tell us we are all wrong, that we are, and indeed have been working contrary to nature's laws, and must turn about and seek new avenues, if we wish for the most good to the greatest number, one thing is certain if they are right, the greatest number are wrong. As for me I have placed the claims of the new departure in the scales, and in my weak judgment, proclaim them wanting, and for that reason I ask, "Where is the Fault?" The title of this essay, gentlemen, reminds me of a prominent lady who has worked zealously for the cause of female suffrage, you will allow me to refer to it. This talented lady was announced to lecture in a neighboring city. All arrangements had been made and a competent man secured to introduce the speaker. She is a prompt woman, and when the time came for her to appear she would not wait for the tardy gentleman, but walked manfully upon the stage and facing a large audience, she was immediately filled with unbounded enthusiasm and stretching her hand heavenwards exclaimed, Ladles and gentlemen, what was I born for? And then in feverish excitement crossed to the end of the stage and raising her hand higher called out in louder tones, What was I born for? Then walked to the other end and called louder than before, ladies and gentlemen, I say, What was I born for? At this juncture an old inebriate back in the audience who had not signed the red ribbon pledge, raised up with an old slouch hat in his hand, and with down cast eyes and sad look, said in a graveyard voice, "Good lady I—I—give it up."

Now gentlemen, although you have not signed the pledge, nor perhaps subscribed to the articles of the new departure don't one of you, when I ask where is the fault say, "I give it up"

That there is a fault not one of us, accepted creed or otherwise, dare deny, but is that fault in mind, or is it in hand, or appliances, or can it be in the material used or is it in the subject worked upon. I stand here to-day, gentlemen, to claim that the fault is not in the appliances, nor in the material, nor as a rule is it in the subject worked upon. I admit in my effort to substantiate this claim I oppose men who have spent more time in research and experiment, than the experience of the average practitioner, but while I am open to conviction I also claim the evidence produced by the opposition insufficient to warrant us in adopting the articles of the new departure, particularly let me insist upon this when I know but a few years since these men stated that gold was pre-eminently the best material for preserving decayed teeth and can we accept this new doctrine upon the basis of only a few years? No, no, gentlemen, let us hold on to that which we know to be good and be wide-awake, read our journals, investigate and judge for ourselves. Gentlemen, while I take issue with the claims of the new departure, I do it not alone through choice but upon conviction based upon seventeen years constant effort, and therefore, take a decided stand against the assertion, that in proportion as teeth need saving gold is the worst material to use; but to the contrary, I believe gold to be the very best material to use, and as mind and hand are applied, so success or failure follows. Here gentlemen, I think is the main fault, as I have said before the fault is not in the appliance, nor material, nor subject, but in the application of mind and hand, and I mean by this the ability to concentrate the mind; force and hand education, coupled with the working qualities of the material in restoring nature's lost tissues. I admit thismay cause a greater physical and mental strain upon some than upon others, as Dr. Flagg said it sapped him, but to such let them do less, for that is no reason why the best should be discarded; but gentlemen, this movement against the noble metal is not the first attack it has received. Sometime between the years of 1849 and 1856, the sponge gold of A. J, Watts was taken hold of with such eagerness by leading operators that gold foil was thought to have seen its day. Sponge gold fillings were displayed with a zest and a new era in operative dentistry seemed certain, and while the inventor of this new material cried out "Eureka," the progressive dentist considered it the *neplus ultra*, but alas—alas, they were doomed to disappointment, and its warmest friends soon learned it was of premature birth and prodigal like, returned to their parent foil; but sponge gold had its virtues, for it led Dr. Arthur to discover that foil had the same cohesive properties and could be manipulated with greater certainty. And this, gentlemen, was indeed a new era in operative dentistry. Here allow me to suggest that perhaps the friends of the new departure may follow in the same path and after a time, return to the material that has the true ring. Oh, did I say the true ring, yes the true for just as you have been true to it so will it be true to you; in other words, if badly introduced and impacted a poor filling will be the result, while if care and ability are bestowed a good and permanent filling will be your reward.

Why gentlemen, so abused is this subject of foil filling that I have heard men say, it required no particular ability nor thinking to fill teeth. I know a man who boasted to me that he perfected a patent bird-trap from which he realized several hundred dollars, while filling teeth. Well it may be a mechanical operation to fill a tooth and it may require mechanical skill to invent and perfect a bird trap, but I do assure you it is impossible to fill teeth with gold foil and catch birds at the same time; and if you do try it the first thing you know you will catch thunder (or ought to), from your abused patient, and the result will be, the filling a failure,—the tooth condemned to second class and you perhaps joined the new departure. I repeat it gentlemen, a perfect gold filling is only secured after proper preparation of the cavity. I mean you should use brains as well as excavators and then the proper use of gold foil, combined with close attention. I will even go farther, and say that you ought not to leave your patient after you have commenced to introduce the gold, that is to wait upon another person, for it dissipates as it were, that beautiful mind structure by dividing or drawing off your close attention, and if you do leave your so far perfect filling, very likely after the operation is completed you will be able to put a pin just where you stopped; for the fingers must have the hearty co-operation of the mind. True enough the fingers may be educated to the key-board of the piano, so that even the blind can play, but not so with the manipulation of gold foil. How very important then, that every operator should train himself to keep his mind upon the operation and not leave it to the care of his fingers? The dentist who does not try under all circumstances to make a perfect filling, is not worthy of being entrusted with the care of these important organs. I feel certain if we would apply more brainism to dental operations, there would be fewer wrecks to attribute to galvanic action, chemical dissolution, etc., how can it be otherwise, just think how small a mistake in the operation of introducing and impacting the foil will result in certain disaster and then compare how much good has been secured where brains and acquired skill have supplied nature with the prevention and the cure. It has been said that of all surgical operations upon the human body there are perhaps none so universally successful as that of filling teeth, for we not only avert disease but restore the lost part of the organ. Dr. Harris said that filling teeth was the most difficult operation the dentist was called upon to perform; that often the skill of the practitioner of ten or twenty years was baffled; but when well and thoroughly performed it was a certain remedy for deep seated caries if afterwards kept thoroughly clean. Dr. Arthur claims a filling capable of accomplishing perfectly the desired end, must be of such material that it will itself undergo no change in the mouth; it must part be capable of being brought in close contact with every of the cavity; it must retain its position immovable; it must be capable of being so dense as to resist the force of mastication; it must be impermeable to the fluids of the mouth; it must be capable of receiving and retaining a perfectly smooth and polished surface. And to gain all these we must rely upon the useful, the beautiful and the wonderful properties of gold foil, it is so tractable that it can be adapted to a nicety to the walls of a cavity, it is incapable of change from contact with agents which are secreted, generated or which come accidently into the mouth; true it has no assistance from nature, but when with that skill becoming intelligence it is indeed an effectual prevention and rendering the tooth artificially sound again. Yes gentlemen, it is difficult to make such a filling, the mind and hand undergoing constant training and education requiring study and effort that the judgment and skill may be equal and for this you have a perfect filling. But how is it with the plastic filling? The editor of the *Register* best expressed my views in an editorial in the August number 1877. He wrote that for the last thirty or forty years the cry has been in the air, "here is the amalgam free from oxydation; will not discolor; will not shrink and will not admit moisture; will save the teeth better than gold and is very easy to manipulate." Now he says we know of no such amalgam, he says these claims are extravagent and present quite a deviation from the truth. Oh, gentlemen here is a substantial difference between the two filling materials in the one just as you bestow skill, judgment and labor so will it remain, while in the other you go it blind and hope there will be no change. I know they call it all mechanical, while the blind one they call chemically preserved and that there is an electrical current established as soon as the gold is introduced, but they very handsomely admit they can't demonstrate its existence. And so, gentlemen, it is with a great deal of the new doctrine, we must accept it on the grounds of reasonableness and consistency and now while speaking of the new doctrine let me add that I do not expect to make an assault upon the views of the gentlemen who have proclaimed the articles of the new departure, only one sentence will I refer to it is, "Mark my words! This is a belief which I am speaking to you. Men are coming to our belief every day—the thinking men, the observing men of dentistry. They write me that they introduce their beautiful jewels of gold, with the view of their premature wreck staring them in the face."

Well gentlemen, I can't express my feeling after reading this verse. It is to me simply awful. What—the thinking, the observing men of dentistry subjecting their patients day after day to the amount of pain and endurance necessary to make a good gold filling with the view of the premature wreck staring them in the face. No wonder their fillings are failures. I care not if a man has been in practice a hundred years, and is capable of entertaining the whole science of dentistry, he can not operate successfully with his fingers when his mind is building up before him a complete wreck. As a comparison let me ask what surgeon would perform a capital operation if he knew his patient would die a few minutes after the operation? I don't want to be understood that it requires faith to do good dental operations, but I do wish to be understood that it requires a degree of certainty coupled with judgment, skill, application and patience to make good and lasting gold fillings—neither do I wish to be understood as questioning Dr. Flagg's statements for I have not the least disposition to doubt his sincerity. He is a personal friend of mine, and I know him to be an honest, intelligent gentleman, but I do say this verse does not accord with the past ten years of my practice, to the contrary I can look on quite a number of healthy mouths free from decayed teeth, with from five to forty gold fillings in them. Gentlemen I know there is a fault, I'll not attempt to show you this nor that or any other, but simply add: Watch and pray lest you fall into temptation.

In the February number of the Missouri *Journal* a query appeared as to the cause of the failure of amalgam filling; in the April number of the same journal, Thomas Fletcher replied and in substance claimed, bad manipulation and an over dose of mercury finding in ten grs. of filling six grs. of mercury, whereas he claimed there should be only one to four—perhaps this is so; but we do most emphatically agree with a little foot note in that journal, it said, "there is a fault somewhere in amalgam fillings which render them as a class much less reliable tooth preservers than good gold fillings, and until this fault is discovered amalgam is not, nor can not be the equal of gold where gold is propeıly used." I was going to say and I believe it is true if plastic filling material had remained in the hidden mysteries of the future, there would be fewer quacks and great many more teeth saved—permanently saved with good and true gold fillings, because of its purity and the ability required to make a perfect filling, educating the mind and hand to the noble and true work, each effort bringing into play new ideas that are a credit to the operator, for you well know to be able to manipulate gold foil correctly is altogether a different task from the use of plastic filling material, in the one is hard effort and constant study with close application, while the other is comparatively easy and if you wish you can mentally make out your patients bill, Now gentlemen, is it not reasonable to claim that in the use of gold a man is developing his mind and training his hands, because of the effort required; while in the use of plastic filling materials a man becomes sluggish in mind and careless in hand and soon forgets the most important points. You are aware ease soon develops carelessness; can a man become intelligent by reading dime novels or can his hand retain a delicate touch in hard manual labor; while to be a successful Dental Physician requires both an educated mind and a delicate touch of the hand. In the May number of the *Cosmos* an article appeared from the pen of Dr. Weld, he says, as usefulness (referring to amalgam) as a material for filling teeth is greater than cohesive foil in hands of dentists who can not or will not exercise the body and mind sufficiently to properly introduce and consolidate gold, and now while I know there are men who advocate plastic fillings to whom this does not apply, yet I believe to the majority it is applicable; true enough plastic fillings have their place for good and so have they for harm. Not more than a month since I was shown an amalgam filling which the man boasted was put in without an instrument. He said the dentist (and I am just ashamed to call him a dentist I wish there was another name for him) just took the filling upon his finger and pressed into the tooth and there it has been in two years. I wish you could have seen it, but the ignorance of the patient was a blessing to the dentist. Oh, what a standard for a respectable profession. No wonder it seems at times almost impossible to raise our noble and worthy profession to the position we so much desire and where is the fault? I believe it is in getting away from the true basis, that basis that will develop the mind and refine the feelings. Gentlemen, let us consider well every step taken before departing from the true, the beautiful and the good; and while we are grateful to the gentlemen who are devoting so much time and labor in experiment and investigation, yet we must not look upon the report of these isolated cases as conclusive. There is no use making a great ado about these experiments; for I have no doubt every one can be refuted with an isolated case from real life. These reports are not sufficient evidence to change us from a standard basis of a half century, but like the sponge gold movement will have its virtue in making us more thoughtful in operating upon these delicate and vascular organs which require both professional skill and mechanical ability. As I have said, we must not be governed by isolated cases. I will here report one as wrong against the new departure as any its advocates have offered against the accepted creed: Mrs. L. Pegan, of our city, came under my care in 1864, when I filled nine cavities with Lawrence's amalgam. According to her statement she commenced droolings soon after the fillings were put in. It gradually increased until she consulted a physician, who, after learning that she had been a subject to ptyalism, supposed that the cause and treated her accordingly, but without the least benefit. She became weak and was considered in poor health. Another physician treated her for general prostration, but yet no relief from this constant waste, which was so bad now that a towel had to be placed under the head on retiring at night, and after being treated by the third physician and enduring the annoyance for more than ten years she came to me to see if I thought her teeth the cause, as she had been told by physician No. 3 that it must be those black fillings in her teeth. I told her it was possible, and accordingly removed them and replaced with gold. She says the drooling was perceptibly less in one week, and now, after three years, there is no return, with no more prostration and general health good.—Now, I ask, judging from this isolated case, is it not consistent and reasonable to claim it dangerous to use amalgam, as for this lady she would think it evil practice; but we all know it will not do to use this isolated case as conclusive. I will report just one more from a long list to show how easy it is to prove gold the only reliable filling material: Miss Liebstadter, a lady of splendid organization, with teeth of good quality. I have had the care of her teeth for a number of years and always used gold, until two years ago she asked for amalgam because her friend said it did not hurt like gold. I have filled five cavities with amalgam, and positively all are failures. Two have not been in twelve months, while the gold fillings are all in excellent condition, doing splendid service. Now suppose we take these two cases to base our judgment upon. In the first case while the amalgam preserved the teeth, it undoubtedly produced such constitutional effects as to warrant us in condemning its use; in the other case, while there was no perceptible constitutional effect it seemed rather to destroy than to preserve the teeth. I say, gentlemen, from these two cases with no rebutting testimony we would be warranted in the claim that amalgam had a dangerous constitutional influence and was a positive failure as a tooth preserver. But we will not attempt to lay claim to such inconsistency, for while amalgam was poison to these two cases it undoubtedly was neat to hundreds. Gentlemen, I have already made my essay longer than I expected, for I prefer fifteen minutes talk to sixty, but let me in conclusion ask your attention to two or three essential points to success in operative dentistry. The first I claim to be kindness and forbearance which are as necessary in the dental physician as the physician in general practice by whose intuitive sympathy the patient is encouraged and often gives to lost vitality one more ray of light which inspires hope. So with the dentist. Many times a patient becomes unmanageable by one cross word or look, when with kindness and forbearance your nervous, trembling patient will submit without complaining. Such an operator will be generally successful. But alas! too few are willing to cultivate such qualities. But remember no man can successfully control others until he has learned to control himself. A man who is so unfeeling that he can not speak a kind word or manifest some spmpathy while exacting of his patient almost inhuman endurance is not adapted to operative dentistry. Undoubtedly it is often through the qualities of kindness, forbearance and sympathy that many painful operations are endured that otherwise would be intolerable. Don't become disgusted because your patient should say it hurts and draws away from your instrument, but for a moment put yourself in their place and I assure you kindness will follow. Tell me who ought be more self-possessed, patient and kind than the operative dentist? It is impossible for a man to manipulate gold foil correctly under mental excitement, and while he is doing all in his power to show kindness to his suffering patient he is not only acting the part of a christian gentleman, but, best of all, is preparing himself for the proper use of mind and hand, and remember he who attempts a gold filling under this excitement is he who really introduces his beautiful jewels of gold with the premature wreck staring him in the face. Here, gentlemen, in this matter of kindness and forbearance, if you stop to examine yourself, you will surely find one of the faults. Another essential point is that your patient should be in a calm, quiet, willing and pleasant condition. But how can they be when the first object they meet is that awful chair, and they do honestly dread the sight of an operating chair. As a rule our dental offices are arranged too much like places of pain, when they might wear the appearance of pleasure. I often wish I could do away with my renowned patent dental chair and substitute an ordinary easy chair that would be rather inviting and free from the repelling influence of that awful chair. And so with the instrument-case. It should be a beautiful piece of furniture, so arranged that every instrument could be kept out of sight, and a general display of pictures, etc., that would carry the mind away from the thought of pain. Tell me, gentlemen, is not pain often suggested at the first sight of some dental offices dark, dirty, ill ventilated, uninviting with a general display of instruments, artificial teeth and even an anatomical specimen under the nose of the trembling patient, whose ideas of anticipated horrors of pain are fully realized before taking the chair, how could you expect a calm enduring patient under such an influence, true some teeth are so sensitive that kindness, forbearance, pleasant rooms, etc., will not suffice: here then let us use plenty of time and the ether spray put upon the rubber dam and after covering the lips and tooth with two folds of the napkin, use the spray with judgment, and I venture you can prepare any cavity, of course it will require patience even with the spray, for the preparation of the cavity is by no means an unimportant part of the labor, the painstaking here will form the foundation and measure the usefulness of the filling. We are apt to treat this operation with too little consideration because of our familiarity from every day effort, intuition, can not fill the minds eye with the finished operation, nor plan for its true construction, this hand work without brains has exposed many healthy pulp and caused many needless failure in operative dentistry. Gentlemen, if we would do our duty, there is plenty of food for thought from the time the patient takes the chair until they leave it, but instead of preparing the cavity in our minds as well as with our hands and restoring lost tissue in mind as well as with instruments We are so apt to trust the tooth to hand education and let the mind go about some other business, but it is not all in the preparation of the cavity; you may have a well prepared cavity margins and surroundings in excellent condition and yet fail to produce a filling; too much labor and pains can not be bestowed upon the introduction and finishing of the filling, so that it will present a firm, unbroken and polished surface, success depending almost as much upon the finishings as the introduction, nearly all fillings which fail, fail at the cervical margin and why is this: not because it is more liable to decay, but for the failure to apply mind and hand. Dr. Garretson, says that the cervical wall is naturally, the least liable to decay particularly if it should reach the cementum providing there is adapted a filling that affords perfect protection. In proof of this, how often have you found decay starting at this place that after being filled proves to be the burden of our cares, but be assured where you have secured a perfect adaptation after a proper preparation of the cervical wall and finally finish your filling, it is in a better condition to resist decay than originally, it is far better where the cavity reaches so far above the margin, of the gum as to occasion a great deal of suffering after the rubber is applied to make sure the cervical wall is well filled and well finished [and then readjust your rubber dam, make new retaining points in the gold and start anew. Now gentlemen, I do unhesitatingly assert that where the application of mind and hand has been used, as I have here spoken of, there will not be seventy per cent failures. I doubt if the failures would amount to more than ten per cent, but with all the kindness and sympathy I have in mind one case that even those qualities will not cure, but where an unflinching determination is the only secret to success. I refer to the patient who directs her dentist not to hurt and if he does she will go to another and while he proceeds with all kindness and care; she continually calling out, Oh! that's enough scraping, fill the tooth and let it go. Now we know dentists are generally human beings and may be influenced to fill the tooth and let it go for fear of losing the patient or it may be we just need that $15 or $20, she will pay, or we don't want her to tell her friends that we are not cruel, etc. But surely and certainly with all kindness we had better be honest with the poor creature and after one honest operation should she choose another, let us say all right, far better to have a clear conscience than a full pocket.

